# Artificial Intelligence in Breast Ultrasound: The Emerging Future of Modern Medicine

**DOI:** 10.7759/cureus.28945

**Published:** 2022-09-08

**Authors:** Srushti S Mahant, Anuj R Varma

**Affiliations:** 1 Department of Medicine, Jawaharlal Nehru Medical College, Datta Meghe Institute of Medical Sciences, Wardha, IND

**Keywords:** computer-aided diagnosis, convolutional neural networks, deep learning, machine learning, artificial intelligence

## Abstract

In today's world, progressively enormous popularity prevails around artificial intelligence (AI). AI is gaining popularity in the identification of various images. Therefore, it has been widely used in the ultrasound of the breast. Furthermore, AI can perform a quantitative evaluation, which further helps maintain the diagnosis's accuracy. Moreover, breast cancer is the most common cancer in women, posing a severe threat to women's health. Hence, its early detection is usually associated with a patient's prognosis. As a result, using AI in breast cancer screening and detection is highly crucial. The concept of AI in the perspective of breast ultrasound has been highlighted in this brief review article. It tends to focus on early AI, i.e., traditional machine learning and deep learning algorithms. Also, the use of AI in ultrasound and the use of it in mammography, magnetic resonance imaging, nuclear medicine imaging, and classification of breast lesions is broadly explained, along with the challenges faced in bringing AI into daily practice.

## Introduction and background

**Figure 1:**
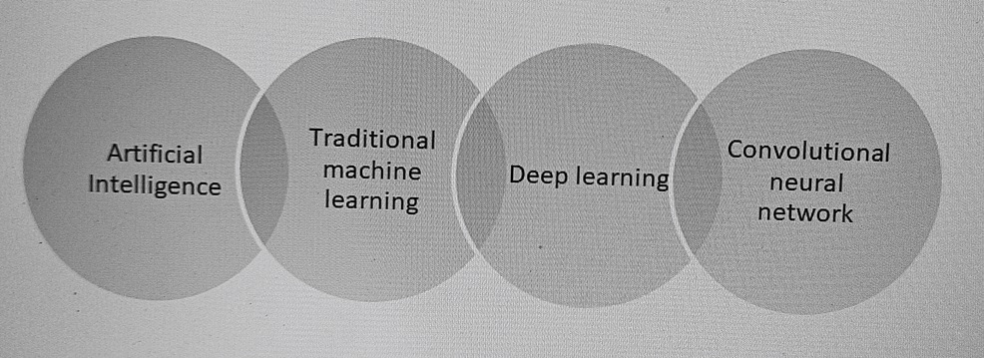
Relationship between the various subsets of artificial intelligence

In women, the most common neoplasm in terms of malignancy is breast cancer. Also, among deaths due to cancer, breast cancer is the second leading cause [1]. By using ultrasound and X-ray, one can diagnose breast cancer. Other significant techniques are mammography and magnetic resonance imaging (MRI), which successfully help make the appropriate diagnosis. First preference in imaging is given to ultrasound for the depiction and categorization of breast lesions as it is non-invasive, feasible, and cost-effective. Along with these, its availability is high and shows acceptable diagnostic performance. Those mentioned above are the basic techniques used as diagnostic tools. Besides these, some newer techniques are available, including color Doppler and contrast-enhanced ultrasound. Spectral Doppler, as well as elastography, also contributes to the diagnosis. These newer techniques support ultrasound doctors to obtain more précised information. However, the drawback is that it does suffer from operator dependence [2]. Deep learning (DL) algorithms, which are precisely a part of artificial intelligence (AI) in particular, have received considerable attention in the past few years due to their outstanding performance in imaging tasks. Technology inbuilt in AI makes better evaluation of the appreciated data related to imaging [3]. AI in ultrasound lays significant focus on distinguishing between benign and malignant masses related to the breast. Radiologists nowadays interpret, analyze, and detect breast images. With a heavy and long-term volume of work, radiologists are more likely to make errors in image interpretation due to exhaustion, which is likely to result in a misidentification or failed diagnosis, which AI can prevent. Humans make various errors in the diagnosis part. To reduce those errors, there is the implementation of a technique known as computer-aided diagnosis (CAD). In this, an algorithm is present that completes the processing of the image along with analysis [4]. Convolutional neural networks (CNNs), a subset of DL, are the most recent technology used in medical imaging [5,6]. AI has the potential to enhance breast imaging interpretation accuracy, speed, and quality. By standardizing and upgrading workflow, minimizing monotonous tasks, and identifying issues, AI has the potential to revolutionize breast imaging. This will most likely free up physician resources that could be used to improve communication with patients and integrate with colleagues. Figure 1 shows the relation between the various subsets of AI in the article.

## Review

Traditional machine learning

Traditional machine learning is the basis and the area of focus that is included under early AI. It deals with the problems in a stepwise manner. It involves a two-step procedure that is object detection followed by object recognition. The first step is object detection, in which case there exists an algorithm for bounding box detection that the machine uses in scanning the image to locate the appropriate object area. The other step, which is the second step, includes the object recognition algorithm that is based on the initial step. Identifying certain characteristic features and encoding the same into a data type are the tasks that experts perform in the identification process. The advantage of a machine is that it extracts the characteristic features, which is followed by performing quantitative analysis, processes the information, and gives the final judgment. In this way, it provides assistance to radiologists in detecting the lesions and analyzing them [5]. Through this, both the efficiency and the accuracy of the diagnosis can be enhanced and improved. In the previous few decades, the popularity of CAD is prevailing in terms of development as well as advancement. CAD includes machine learning methodologies along with multidisciplinary understanding as well as techniques. Analyzing the information of the patient is done by using these techniques. Similarly, the results can provide assistance to clinicians in the process of making an accurate diagnosis [7]. CAD could very well evaluate imaging data. It directly provides the information after analyzing it to the clinician and also correlates the results with some diseases that involve the use of statistical modelling of previous cases in the population. It has many other applications, such as lesion detection along with characterization and staging of cancer, including the enactment of a proper treatment plan with the assessment of its response. Adding to it, prediction of prognosis and recurrence are some more applications. DL has transformed computer vision [8,9].

Deep learning algorithms

DL, which is an advanced form of machine learning, does not depend solely on features and ROIs (region of interest) that are preset by humans, which is the opposite of traditional machine learning algorithms [9,10]. Along with this, it prefers to complete all the targeted processes independently. CNNs are the evolving configuration in healthcare, which is a part of DL. It can be explained by an example. Majorly, the model consists of three layers: input layers, hidden layers, and output layers. In this case, the hidden layer is the most vital determinant in achieving recognition. Being the most crucial determinant of achieving recognition, a significant number of convolutional layers, along with a fully connected layer, are encompassed in the hidden layers. The various massive problems generated by the machine based on input activity are handled by the convolutional layers. They are connected to form a complex system with the help of convolution layers, and, hence, it can easily output the results [11,12]. DL methods have an excessive dependence on data and hardware. In spite of this, it has easily defeated other frameworks in computer vision completion [13]. Furthermore, DL methods perform flawlessly not only in ultrasound but also in computed tomography (CT) [14,15]. According to certain studies, it has also been shown that there has been an adequate performance of DL methods in MRI [16]. DL uses a deep neural network architecture to transform the input information into multiple layers of abstraction and thereby discover the data representations [17]. The deep neural network's multiple layers of weights are iteratively updated with a large dataset with effective functioning. This yields a mathematical model of a complex type that is capable of extracting relevant features from input data showing high selectivity. DL has made major advances in many tasks such as target identification, including characterization, speech and text recognition, and face recognition. Some other advancements are smart devices and robotics.

Artificial intelligence equipped with ultrasound

An ultrasonic machine is used to upload images taken to the workstation, where they are reprocessed. The DL technique (S-detect) can, on the other hand, directly pinpoint breast lesions on the ultrasound. It is also used in segmentation, feature analysis, and depictions. The BI-RADS (Breast Imaging-Reporting and Data System) 2013 lexicon may also be used for the same. It can provide instantaneous results in the form of a frozen image on an ultrasound machine to detect any malignancy. This is performed by selecting ROI automatically or by manual means [18]. They assessed the performance of S-detect in terms of diagnosis so as to confirm whether the breast lesion was benign or malignant. On setting the cutoff at category 4a in BI-RADS, it was observed that the accuracy, along with specificity and PPV (positive predictive value), was high in S-detect in comparison with the radiologist (p = 0.05 for all). Ultrasonologists typically use macroscopic and microscopic features of breast images to recognize and segment potentially malicious lesions. Shape and edge, including orientation and accurate location of calcification, can be detected. Certain features, such as rear type and echo type, along with hardness, can also be identified. Following that, suspicious masses are classified using the BI-RADS scale so as to assess and estimate the level of cancer speculation in breast lesions. However, its macroscopic and microscopic characteristics are critical in distinguishing whether the masses are of malignant type. As a result, ultrasound experts are in high demand for correctly obtaining these features.

Mammographic application of artificial intelligence

Mammography is a non-invasive technique with high resolution that is commonly used. It also shows good repeatability. Mammography detects those masses that doctors fail to palpate in the breasts and can reliably distinguish whether the lesions are benign or malignant. Mammograms are retrieved from digital mammography (DM). They are possibly provided to process (raw imaging data) as well as to present (a post-treated form of the raw data) image layouts using DM systems [19]. Breast calcification appears on mammography as narrow white spots, which are breast calcifications caused by narrow deposits of calcium salts in the tissues of the breast. Calcification is classified into two types: microcalcifications and macrocalcifications. The large, along with rough, are macrocalcifications; they are usually benign and depend on the age group. Microcalcifications, which range in size from 0.1 mm to 1 mm, can be found within or outside visible masses and may act as early warning signs of breast cancer [20]. Nowadays, significant CAD systems are progressing to detect calcifications in mammography.

MRI application of artificial intelligence

DL, like DM, DBT (digital breast tomosynthesis), and USG (ultrasonography), is primarily utilized in MRI to conduct or assist in the categorization and identification of breast lesions. The other modalities and MRI. differ in their dimensions. MRI produces 3D scans; unlike it, 2D images are formed by other modalities such as DM, DBT, and USG. Furthermore, MRI observes the input along with the outflow of contrast agents (dynamic contrast-enhanced MRI) and changes its pre-existing dimensions to 4D. Moreover, hurdles are created while applying DL models on the 3D or 4D scans because the majority of models are designed to function on 2D pictures. To address these issues, various ways have been proposed. The most frequent method is to convert 3D photos to 2D images. It is accomplished by means of slicing, in which the 3D image is sliced into 2D, or by applying the highest intensity projection (MIP) [21,22]. DL is utilized to classify the axillary group of lymph node metastases in addition to lesion categorization [23-25]. Instead of biopsy data, positron emission tomography (PET) is used as the gold standard. The reason is that, while a biopsy is conclusive as truth, it leaves artifacts such as needle marks along with biopsy clips, which may unintentionally lead to shifting of the DL algorithm toward a malignant categorization [23,24].

Nuclear medicine imaging application of artificial intelligence

PET or scintigraphy is a nuclear medicine imaging technique. They are predicted to be not much more suitable than the other previously stated imaging modalities, namely DM, digital tomosynthesis, USG, and MRI, for evaluating early-stage of cancerous lesions in the breast. The nuclear techniques, on the other hand, provide added utility for detecting and classifying axillary lymph nodes along with distant staging [26]. As a result, it is not surprising that DL is being used in this imaging field, albeit in a limited capacity. PET/CT assessment of whole-body metabolic tumor volume (MTV) could provide a measure of tumor burden. If a DL model could handle this operation, it would considerably minimize manual labor because, in practical application, for acquiring MTV, all tumors must be identified [27,28]. Weber et al. investigated whether a CNN trained to detect and segment the lesions in the breast with whole-body PET/CT scans of patients who have cancer could also detect and segment lesions in lymphoma and lung cancer patients. Moreover, the technique of DL, along with nuclear medicine techniques, are used parallelly in improving the tasks that are similarly used in other imaging approaches. Li et al. developed a 3D CNN model to help doctors detect axillary lymph node metastases on PET/CT scans [28]. Because of their network, clinicians' sensitivity grew by 7.8% on average, while their specificity remained unchanged (99.0%). However, both clinicians outscored the DL model on its own.

Classification of breast lesions

Worldwide, it has been observed that in women, there is a higher incidence and fatality rate of breast cancer; hence, many countries have implemented screening centers for women of the appropriate age group for detection of breast cancer. The ideology behind the implementation of screening centers is to distinguish between benign breast lesions and malignant breast lesions. The primary classification system used is BI-RADS for classifying lesions in breast ultrasound. AI systems have been developed with equipped features for classifying benign and malignant breast lesions to assist clinicians in making consistent and accurate decisions. Ciritsis et al. categorized breast ultrasound images into BI-RADS 2-3 and BI-RADS 4-5 using a deep convolution neural network (dCNN) with an internal data set and an external data set. The dCNN had a classification accuracy of 93.1% (external 95.3%), whereas radiologists had a classification accuracy of 91.65% (external 94.1 2%). This indicates that deep neural networks (dCNNs) can be utilized to simulate human decision-making. Becker et al. analyzed 637 breast ultrasound pictures using DL software (84 malignant and 553 benign lesions). The software was trained on a randomly chosen subset of the photographs (n=445, 70%), with the remaining samples (n=192) used to validate the resulting model during the training process. The findings demonstrated that the neural network, which had only been trained on a few hundred examples, had the same accuracy as a radiologist's reading. The neural network outperformed a trained medical student with the same training data set [29-31]. This finding implies that AI-assisted classification and diagnosis of breast illnesses can significantly cut diagnostic time and improve diagnostic accuracy among novice doctors. Table 1 shows BIRADS scoring.

**Table 1:** BIRADS score BIRADS, Breast Imaging Reporting and Data System

BIRADS score	Category	Management	Risk of cancer
0 (incomplete investigation)	Need additional imaging or prior examination	Recall additional imaging or await prior examination	n/a
1	Negative	Routine screening	Essentially 0%
2	Benign	Routine screening	Essentially 0%
3	Probably benign	Short interval follow-up (6 months) or continued	>0% but <2%
4	Suspicious	Tissue diagnosis	4a. low Suspicion for malignancy (>2% to <10%). 4b. Moderate suspicion for malignancy (>10% to <50%). 4c. High suspicion for malignancy (>50% to <95%)
5	Highly suggestive of malignancy	Tissue diagnosis	>95%
6	Biopsy-proven malignancy	Surgical excision when clinically appropriate	n/a

Challenges

AI is still struggling to advance to a higher level. Although it is tremendously progressing in the healthcare fraternity, it still has to cover a long journey to properly blend into clinicians' work and be widely implemented around the world. Many limitations have been reported for CAD systems for breast cancer screening, including a global shortage of public datasets, a high reliance on ROI annotation, increased image standards in terms of quality, regional discrepancies, and struggles in binary classification. Furthermore, AI is designed for single-task training and cannot focus on multiple tasks at once, and, hence, it is one of the significant obstacles to the advancement of DL associated with breast imaging. CAD systems are progressively evolving in ultrasound elastography [32]. Similarly, it is also progressing in the technology related to contrast-enhanced mammography as well as MRI [33,34]. AI in breast imaging can be used to not only detect but also classify breast diseases and anticipate lymph node tumor progression [35]. Moreover, it can also predict disease recurrence. As the technology of AI advances, there will be higher accuracy, along with greater efficiency, and a more precise plan of treatment for breast ailments, enabling them to achieve early detection with accurate diagnosis among radiologists. Moreover, it can also predict disease recurrence. The lack of a consistent strategy for segmentation (2D vs. 3D), feature extraction, and selection and categorization of significant radiomic data is a common limitation shared by all imaging modalities. Future studies with greater datasets will allow for subgroup analysis by patient group and tumor type [36].

## Conclusions

Without a crystal ball, it is impossible to predict whether further advances in AI will one day start replacing radiologists or other functions in diagnostics reportedly performed by humans, but AI will undeniably play a major role in radiology, one that is currently unfolding rapidly. When compared to traditional clinical models, AI has the added benefit of being able to pinpoint distinctive features, textures, and details that radiologists seem unable to appreciate, as well as quantitatively define image explicit details, making its evaluation more objective. Moreover, AI in breast imaging can be used to not only detect but also classify breast diseases. As a result, greater emphasis needs to be placed on higher-quality research studies that have the potential to influence treatment, outcomes for patients, and social impact.
